# The effects of spinal manipulation on performance-related outcomes in healthy asymptomatic adult population: a systematic review of best evidence

**DOI:** 10.1186/s12998-019-0246-y

**Published:** 2019-06-07

**Authors:** Melissa Corso, Silvano A. Mior, Sarah Batley, Taylor Tuff, Sophia da Silva-Oolup, Scott Howitt, John Srbely

**Affiliations:** 1 Canadian Memorial Chiropractic College, 6100 Leslie Street, North York, ON M2H 3J1 Canada; 2 University of Guelph, Guelph, ON Canada

**Keywords:** Spinal manipulation, Athlete, Asymptomatic, Healthy, Performance, Sport

## Abstract

**Introduction:**

The effectiveness of spinal manipulative therapy (SMT) for improving athletic performance in healthy athletes is unclear. Assessing the effect of SMT on other performance outcomes in asymptomatic populations may provide insight into the management of athletes where direct evidence may not be available. Our objective was to systematically review the literature on the effect of SMT on performance-related outcomes in asymptomatic adults.

**Methods:**

MEDLINE, CINAHL, SPORTDiscus, and Cochrane Central Register of Controlled Trials were systematically searched from 1990 to March 23, 2018. Inclusion criteria was any study examining a performance-related outcome of SMT in asymptomatic adults. Methodological quality was assessed using the SIGN criteria. Studies with a low risk of bias were considered scientifically admissible for a best evidence synthesis. We calculated the between group mean change and 95% confidence intervals.

**Results:**

Of 1415 articles screened, 20 studies had low risk of bias, seven were randomized crossover trials, 10 were randomized controlled trials (RCT) and three were RCT pilot trials. Four studies showed SMT had no effect on physiological parameters at rest or during exercise. There was no effect of SMT on scapular kinematics or transversus abdominus thickness. Three studies identified changes in muscle activation of the upper or lower limb, compared to two that did not. Five studies showed changes in range of motion (ROM). One study showed an increase lumbar proprioception and two identified changes in baropodometric variables after SMT. Sport-specific studies show no effect of SMT except for a small increase in basketball free-throw accuracy.

**Conclusion:**

The preponderance of evidence suggests that SMT in comparison to sham or other interventions does not enhance performance-based outcomes in asymptomatic adult population. All studies are exploratory with immediate effects. In the few studies suggesting a positive immediate effect, the importance of such change is uncertain. Further high-quality performance specific studies are required to confirm these preliminary findings.

**Electronic supplementary material:**

The online version of this article (10.1186/s12998-019-0246-y) contains supplementary material, which is available to authorized users.

## Introduction

Chiropractors are an integral part of injury management for the athlete. Fellows of the Royal College of Chiropractic Sports Sciences (Canada) (RCCSS(C)) report that up to 25% of their practice is comprised of athletes and 49.5% of Australian chiropractors report ‘often’ treating athletes [1, 2]. In addition, 29% of intercollegiate athletes use chiropractic treatment [3] and 31% of NFL teams employ chiropractors as an official part of their staff [4]. SMT is a well-documented intervention, typically provided by a chiropractor, for spinal pain and musculoskeletal injuries [1, 3, 5–7]. However, 84% of RCCSS(C) fellows also reported witnessing performance enhancements in asymptomatic athletes immediately after SMT and/or adjunctive therapy [1]. Minor enhancements in performance can have significant implications to an athlete. For example, the difference between first and third place in the 100-m sprint during the 2016 Rio Olympics was only 100 milliseconds [8]. Some athletes insist on being treated before competition to optimize performance despite having no musculoskeletal complaints, and 94% of those athletes utilizing chiropractic care at the 2013 World Games reported immediate improvements [9].

Despite the anecdotal evidence that SMT improves performance, the literature on the effect of SMT on athletic performance and/or performance-related outcomes is equivocal and lacks methodological rigor. Miners [10] conducted a narrative review and reported that, although many theoretical frameworks have been proposed, insufficient evidence exists to support the claim that chiropractic treatment directly and significantly improves performance in athletes. The theoretical frameworks identified by Miners [10] include the improvements of abnormal spinal mechanics, muscular coordination, activation and reaction time, and motor training [10]. In 2017, Cerqueira et al. [11] and Botelho et al. [12] conducted similar but separate systematic reviews assessing the effect of SMT on performance in athletes, with the Botelho et al. review including three additional studies. Cerqueira et al. [11] concluded that current evidence is insufficient to determine the use or non-use of SMT to improve athletic or sport-specific performance [11]. In contrast, while Botelho et al. [12] agreed that while the evidence is weak, most studies showed an improvement in athletic performance with SMT [12]. Both authors recommend that this topic requires greater methodological rigor and further investigation [11, 12].

All three of these reviews were specific to athletic populations and sport-specific outcomes. Limitations of these reviews include lack of clear inclusion criteria for studies and inclusion of studies of low methodological quality. Furthermore, these reviews did not include studies measuring the effect of SMT on performance outcomes among asymptomatic populations that may be relevant to athletic performance. Such performance effects might also be of interest to athletes who are seeking performance benefits, as they would be receiving SMT in the absence of pain or injury, similar to an asymptomatic subject. Both of these populations, whether an asymptomatic healthy subject or an athlete seeking performance enhancement would be expected to fundamentally respond in the same direction when stimulated by an external force such as SMT [13, 14]. However, the application of this response would differ between the populations, for example, a sprint in the athlete or walking ability in a non-athlete. We suggest that the response to SMT in either population would be similar, while the context of its application changes based on the tasks required of the subject. Thus, our systematic review includes studies investigating healthy asymptomatic adult populations for related measurable changes that may directly or indirectly affect performance and/or function rather than to manage specific injuries or neuromusculoskeletal complaints.

Our objective was to systematically review the literature on the effect of SMT compared to other interventions, placebo/sham intervention, and no intervention on performance-related outcomes (ie, physiological, biomechanical, and sport-specific outcomes) in healthy asymptomatic individuals.

## Methods

### Registration

We registered this review protocol with the International Prospective Register of Systematic Reviews (PROSPERO) on May 22, 2017 (CRD42017067090).

### Search strategy

We developed our search strategy in consultation with a health sciences librarian, and the strategy was reviewed by a second librarian using the Peer Review of Electronic Search Strategies (PRESS) Checklist [15, 16]. The following electronic databases were systematically searched from January 1, 1990 to March 23, 2018: MEDLINE, CINAHL, SPORTDiscus, and Cochrane Central Register of Controlled Trials. Search terms consisted of subject headings specific to each database (e.g., MeSH in MEDLINE) and free text words relevant to our PICO components (Additional file 1: Appendix I). In addition, we hand searched the reference lists of relevant studies for any relevant citations that may not have been retrieved by our search.

### Selection of studies

Studies were included if they met the following criteria: 1) published in English language and in a peer-reviewed journal; 2) study designs including: randomized controlled trials (RCT), cohort, and pilot studies; 3) study population including asymptomatic adults (> 18 years old); 4) intervention consisted of spinal and pelvic SMT (high velocity, low amplitude) compared with placebo/sham, no intervention, or an alternative intervention; and 5) outcome measures must include at least one performance parameter such as range of motion, strength, power, motor control, agility, balance, or speed.

Studies fulfilling any of the following criteria were excluded: 1) publication types including: guidelines, letters, editorials, commentaries, unpublished manuscripts, dissertations, government reports, books and book chapters, conference proceedings, meeting abstracts, lectures and addresses, consensus development statements, guideline statements; 2) study designs including: feasibility and cross-sectional studies, case reports, case series, qualitative studies, non-systematic and systematic reviews, clinical practice guidelines, studies not reporting on methodology; and 3) cadaveric or animal studies. We used the National Institute for Health Research (NIHR) definitions to distinguish pilot and feasibility studies; pilot studies are smaller versions of the main study whereas a feasibility study are used to estimate important parameters that are needed to design the main study [17].

### Definitions

SMT was defined as a controlled, high-velocity, low amplitude mechanical intervention leading to the deformation of the spine and surrounding soft tissues [18]. Athletic performance, defined by the National Strength and Conditioning Association, is the ability to respond effectively to various physical challenges [19]. Examples of features related to athletic performance include, but not limited to: strength, power, endurance, agility and speed [19]. Biomechanics is defined as the study of biological systems, particularly of their structure and function, and specifically being concerned with the effect these forces have on motion of bodies [20]. Physiology is defined as the study of the functioning of living organisms (animal or plant) and of their constituent tissues or cells [21]. Asymptomatic is defined as a study population presenting with no current symptoms of disease [22].

### Screening of titles and abstracts

All citations identified by the search strategy were exported into EndNote X6 for reference management and tracking of the screening process. We used a two-phase approach to screening with pairs of independent reviewers screening each citation and article (MC with SM, TT, SH, JS). In the first phase, titles and abstracts were screened for relevant, irrelevant and possibly relevant citations. Possibly relevant citations from the first phase were reviewed in the second phase using full text screening. Any disagreement was resolved by discussion between the paired reviewers to reach consensus. If consensus was not reached, a third reviewer independently appraised the citation and discussed with the other two reviewers to reach consensus.

### Critical appraisal

All relevant studies were critically appraised for risk of bias independently by rotating pairs of reviewers (MC with SM, SB, TT, SD, SH, JS). Risk of bias was assessed using the Scottish Intercollegiate Guidelines Network (SIGN) criteria for randomized controlled trials and cohort studies [23, 24]. The SIGN criteria were used to evaluate the nature and impact of selection and information bias, and potential confounding on the results of the study. A quantitative score or cut-point was not used to determine internal validity. The SIGN criteria guided reviewers in their assessment of the overall internal validity of the study, and studies scored as “accepted” were included in the final analysis [25].

We critically assessed the following methodological aspects (where appropriate or applicable) in each study: clarity of research question; randomization method; concealment of the treatment allocation; blinding of treatment and outcomes; similarity of baseline characteristics between/among treatment arms; co-intervention contamination; validity and reliably of outcome measures; follow-up rates; analysis according to intention-to- treat principles; and comparability of results across study sites.

Following critical appraisal, studies with a low risk of bias as determined and agreed upon by reviewing pairs were considered scientifically admissible for a best evidence synthesis [26]. We did not exclude studies based on a minimum number of participants as suggested by others [6]; rather the precision of the study’s findings was considered when interpreting the results and conclusion by assessing the between group mean change and 95% confidence intervals (CI) where possible. We did not conduct a sensitivity analysis as this was considered beyond the scope of this review.

### Synthesis

One reviewer (MC) extracted data from scientifically admissible studies to build evidence tables (Table 1). Data extraction was checked by a second reviewer (SM). Evidence tables outlined the calculated between group mean change and 95% CI where applicable, best evidence on each topic, identified consistencies and inconsistencies in the evidence and were used to create summary statements describing the body of evidence [26]. Evidence was stratified based on outcome measures into one of three categories: physiological, biomechanical and sport-specific outcomes.

**Table 1:** Evidence table of included studies, in alphabetical order

Authors, Year	Subjects & Setting; # enrolled; Design	Interventions; # subjects	Comparisons; # subjects	Follow-Up	Outcomes	Key Findings Mean change (95% CI)
Physiological Outcomes						
Budgell B and Polus B, 2006 [30]	No current neck or upper back pain; 18–45 years; *n* = 28; Japan; Controlled crossover trial, 1 week apart.	PA thrust SMT to upper thoracic spine (1–4 vertebral levels) dependent on motion restriction. (cross-bilateral or combination SMT).	Sham: hands over the scapulae bilaterally, with a single light brief impulse simultaneously with both hands.	Immediately post intervention.	ECG recording for 5-min blocks pre-SMT and post-SMT. Adverse events.	Mean differences between groups SMT-Sham: HR: − 0.24 bpm (− 4.15, 3.67) LFab: − 50.5 (− 126.09, 25.09) LFn: − 4.99 (− 11.54, 1.56) HFab: 122.2 (− 242.49, 486.89) HFn: 4.43 (− 2.22, 11.08) LF/HF: − 0.2578 (− 0.61, 0.09) Adverse events: Sham: 1 subject, 3.8/10 on VAS. SM: 2 subjects, 1.3 and 1.4/10 on VAS.
Da Silva et al., 2013 [33]	Healthy university students, no regular physical activity; 20–30 years; Brazil; *n* = 67; Single-blind placebo-controlled clinical trial.	Cervical SMT group: supine rotary SMT of C3. *n* = 15 Thoracic SMT group: side-lying rotary thrust of T12. *n* = 15 Cervical and thoracic SMT group: cervical SMT followed by thoracic SM. n = 15	Anterior tibiotarsal mobilization: AP glide of the tibia on the talus. *n* = 14	Immediately post intervention.	Maximum inspiratory pressure (MIP), maximum expiratory pressure (MEP), total lung capacity (TLC) and residual volume (RV).	N.S. between-group differences between groups. *Unable to calculate mean change and 95% CI.
Ward J, 2013 [47]	Apparently healthy chiropractic students; 20–29 years; Texas, USA; *n* = 20. Single-blind, RCT.	Side-posture mammillary push at L3, performed bilaterally. *n* = 10	No SMT. n = 10	HR and Rate of Perceived Exertion (RPE): at the conclusion of each 3 min exercise test stage; Blood lactate concentration (BLC): conclusion of the exercise test. Time to exhaustion: at conclusion of the test.	HR (bpm), RPE (Borg scale), BLC, time to perceived exertion and VO2 max (calculated from time to perceived exertion) during the Bruce treadmill protocol.	Velocity response 4 mmol/L: 0.1 mph (−0.27, 0.46) Velocity response 8 mmol/L: 0.1 mph (− 0.36, 0.57) HR 4 mmol/L: 2.9 bpm (− 8.81, 2.91) HR 8 mmol/L: 4.8 bpm (− 4.18, 13.88) RPE 5mph: − 0.3 (− 1.99, 1.39) RPE 6mph: − 0.1 (− 2.22, 2.02) RPE 7mph: 0.1 (− 2.59, 2.79) RPE 8mph: − 1 (− 6.07, 4.07)
Biomechanical Outcomes: Electromyography/Muscle Activation						
Christiansen et al., 2018 [31]	Elite level Taekwondo athletes with subclinical neck pain; 17–50 years; Auckland, New Zealand; *n* = 12; Within-subject randomized controlled crossover trial, 1 week apart.	SMT HVLA thrust to areas of segmental dysfunction throughout entire spine and SIJs. *n* = 11	Head and spine moved passively and actively similar to SMT without HVLA thrust.	Immediately, 30 min and 60 min post intervention.	Surface EMG of plantarflexors during maximum voluntary contraction (MVC) (% change).	MVC between group % change SMT-Sham: Immediately post: 11.09% (3.63, 18.55) 30 min post: 13.68% (4.51, 22.85) 60 min post: 9.74% (0.79, 18.59)
Dunning et al., 2009 [35]	Asymptomatic, physiotherapy and nursing students; 18–40 years; Birmingham, UK; *n* = 54; Crossover RCT with 8 min of washout.	HVLA rotary SMT to the right C5/6 segment.	Sham SMT to the right C5/6 segment. Control: no manual contact for 30 s.	Immediately post intervention.	Resting EMG (rEMG) of the biceps brachii muscles (mean % change).	Between group mean % change: Right: SMT-control: 98.38% (84.08,112.68) SMT-sham: 73.08% (59.43, 86.73) Sham-control: 25.30% (19.63, 30.97) Left: SMT-control: 82.19% (67.06, 97.33) SMT-sham: 62.89% (49.18–76.59) Sham-control: 19.31% (10.10–28.52) S.S. greater change of the right compared to the left: (14.16%)
Grindstaff et al., 2009 [38]	Healthy adults, asymptomatic last 6 months; 18–37 years; USA; *n* = 42; RCT.	Supine lumbopelvic SMT (high grade mobilization). n = 15	Side-lying lumbar mid-range flexion/extension PROM for 1 min, lower grade joint mobilization. *n* = 13. Lying prone on elbows for 3 min, sham treatment. n = 13.	Immediately after and 20, 40, and 60 min post-intervention.	Quadriceps maximal voluntary isometric contraction (MVIC) (% change) and central activation ratio (CAR) (% change).	MVIC: SMT-PROM = 8.1% (5.52, 19.44) SMT-Prone extension = 12.1% (1.36, 15.28) CAR: SMT-PROM = 5.0% (0.37, 9.92) SMT-Prone extension = 6.5% (1.34, 10.90) N.S. change in % change MVIC or CAR from baseline at 20, 40 or 60 min. *Unable to calculate mean change and 95% CI
Pollard et al., 1996 [13]	Healthy chiropractic students; 18–40 years; Sydney NSW; *n* = 30; Controlled before/after intervention study.	Lumbar roll position with SMT of the right L3/4 segment. n = 15	Sham: Simulated SMT to the left side of the L3/4 motion segment while in the lumbar roll position. n = 15	Immediate after SMT	Average of the force of two maximal isometric contractions of the quadriceps femoris (N).	Between group difference SMT-Control: 5.1 N (− 3.67, 13.87)
Sanders et al., 2015 [46]	Healthy, asymptomatic, never received SMT; 20–35 years; Kentucky, USA; *n* = 21; Randomized, controlled, single-blind crossover design.	Bilateral side-lying lumbar and/or SIJ SMT to identified restrictions. n = 21	Sham: use of drop piece and non-specific thrust through lumbar paraspinals. n = 21	Within 5 min post-treatment, and again after 20 min.	Maximal voluntary isometric contractions (MVIC) of extension and flexion at 60° knee flexion. Isokinetic, concentric MVIC of knee extension and flexion at 60°s and 180°/s (% change).	Between group SMT-Sham % change extension & flexion at 60° knee flexion: 2.8% (− 2.23, 7.83) Between group SMT-Sham % change isokinetic contractions at 60°/s: − 3.7% (− 10.93, 3.53) Between group SMT-Sham % change isokinetic contractions at 180°/s: 4.1% (− 6.64, 14.84)
Biomechanical Outcomes: Range of Motion						
Galindez-Ibarbengoetxea et al., 2017 [36]	Asymptomatic participants, 18–40 years; Spain; *n* = 36; Prospective, randomized controlled pilot study.	AMC5 group: HVLA to right C5. n = 12 MT group: Joint dysfunction of cervical and thoracic spine evaluated and HVLA as needed. n = 12	ST group: same protocol as AMC5 but 3 rotation movements without reaching barrier. n = 12	Immediately post intervention.	Cervical spine ROM (°), cervical flexion isometric peak force (N), surface EMG of SCM (mV), cervical erector spinae and biceps brachii (mV). Adverse events.	Cervical extension ROM: AMC5-MT: 2.5° (− 3.87, 8.87) AMC5-ST: 7.3° (1.02, 13.58,) MT-ST: 9.8° (5.18, 14.42) Cervical flexion ROM: AMC5-MT: 0.09° (− 5.94, 6.12) AMC5-ST: 2.41° (− 3.73, 8.55) MT-ST: 2.5° (− 3.58, 8.58) Cervical right lateral flexion ROM: AMC5-MT: 1.25° (− 3.89, 6.39) AMC5-ST:- 0.91° (− 7.01, 5.19) MT-ST: 29.8° (24.36, 35.24,) Cervical left lateral flexion ROM: AMC5-MT: 1.83° (− 3.3, 6.96) AMC5-ST: − 1.83° (− 7.49, 3.83) MT-ST: 0° (− 4.79, 4.79) Cervical right rotation ROM: AMC5-MT: − 2.83° (− 9.07, 3.41) AMC5-ST: 4° (− 1.62, 9.62) MT-ST: 1.17° (− 3.58, 5.92) Cervical left rotation ROM: AMC5-MT: 3.66° (− 1.94, 9.26) AMC5-ST: 0.1° (− 5.97, 6.17) MT-ST: 3.76° (− 1.10, 8.62) Cervical flexion isometric peak force: AMC5-MT: − 2.76 N (− 8.1, 2.58) AMC5-ST: 0.47 N (− 4.22, 5.16) MT-ST: 3.23 N (− 2.63, 9.09) Biceps brachii EMG at rest: AMC5-MT: Right − 35.07 mV (− 80.85, 10.71) Left − 3.76 mV (− 53.64, 46.12) AMC5-ST: Right − 36.9 mV (− 90.91, 17.11) Left 121.68 mV (56.00, 187.36) MT-ST: Right − 1.83 mV (− 60.55,56.89) Left 125.44 mV (59.94, 190.94) SCM during cranio-cervical flexion test: AMC5-MT: Right − 7.2 mV (− 18.91, 4.51) Left 2.47 mV (− 10.25, 15.19) AMC5-ST: Right 8.69 mV (− 4.95, 22.33) Left 4.35 mV (− 8.51,17.21) MT-ST: Right 15.89 mV (1.79, 29.99) Left 1.88 mV (− 11.51, 15.27) No adverse events.
Gavin D, 1999 [37]	Asymptomatic; 22–44 years; USA; *n* = 78; Single-blind RCT.	Group 3: SMT group, supine or seated SMT to hypomobile segments of the thoracic spine and ribs. *n* = 26	Group 1: control group, waited 4 min in a separate room with the manipulating therapist. n = 26 Group 2: mobility group, prone segmental mobility test from T3-T8. n = 26	Immediately post intervention.	Thoracic spine (T3-T8) seated AROM (°) in forward bend, right & left side bend.	Forward bending ROM: Control-Palpation: − 1.1 ° (− 2.50, 0.30) SMT-Control: 1.0° (− 0.47, 2.47) SMT-Palpation: − 0.1° (− 1.83, 1.63) Right-side bend ROM: Control-Palpation: − 0.3° (− 2.09, 1.49) SMT-Control: 1.5° (0.17, 3.17) SMT-Palpation: 1.2° (− 0.64, 3.04) Left-side bend ROM: Control-Palpation: − 0.7° (− 2.13, 0.73) SMT-Control: 2.2° (0.91, 3.49) SMT-Palpation: 1.5° (− 0.18, 3.18)
Hanney et al., 2017 [39]	Students, faculty or staff of University of Central Florida; 18–50 years; Florida, USA; *n* = 102; Randomized controlled trial.	Bilateral cervicothoracic thrust manipulation. *n* = 34	Manual stretching: supine with passive flexion, lateral flexion away and rotation of head towards stretched side until barrier was met for 30 s, 2x/side. n = 34 No treatment: seated for 3–5 min. n = 34	Immediately after intervention.	Cervical ROM (flexion, extension, bilateral lateral flexion and rotation) (°).	S.S. group x time interaction: cervical extension ROM and bilateral lateral flexion. Extension ROM: SMT-Control: 3.76° (− 0.37, 7.15) SMT-Stretch: − 4.29° (− 7.72, − 0.86) Control-Stretch: − 8.05° (− 11.42, − 4.68) L lateral flexion ROM: SMT-Control: 2.82° (0.18, 5.46) SMT-Stretch: 0.35° (− 2.06, 2.76) Control-Stretch: − 2.47° (− 5.26, 0.32) R lateral flexion ROM: SMT-Control: 3.76° (0.94, 6.58) SMT-Stretch: − 0.47° (− 3.31, 2.37) Control-Stretch: − 4.23° (− 7.26, − 1.20) Cervical flexion ROM: SMT-Control: 3.61° (0.16, 7.06) SMT-Stretch: − 0.65° (− 3.86, 2.56) Control-Stretch: − 4.26° (− 7.58, − 0.94) Left cervical rotation ROM: SMT-Control: 3.0° (0.38, 5.62) SMT-Stretch: 0.53° (− 1.70, 2.76) Control-Stretch: − 2.47° (− 5.04, − 0.10) Right cervical rotation ROM: SMT-Control: 1.88° (− 0.70, 4.46) SMT-Stretch: 0.88° (− 1.23, 2.99) Control-Stretch: − 1.0° (− 3.77, 1.77)
Biomechanical Outcomes: Other						
Ditcharles et al., 2017 [34]	Right-handed young healthy adults; 24–32 years; France; *n* = 22; Randomized controlled trial.	Standing “lift-off” technique HVLA SMT to T9. n = 11	Sham: same experimental protocol as HVLA group using “light touch methodology”, without compression or traction. n = 11	Immediately post intervention.	Gait initiation variables: anticipatory postural adjustments (APA) duration (sec), peak of anticipatory backward center of pressure (COP) displacement (m), center of gravity (COG) velocity at toe-off (TO) (m/s), mechanical efficiency of APA (ratio), peak of COG velocity (m/s), step length (m), and swing phase duration (msec); thoracic spine ROM (°).	N.S. main effect of group x condition for spine ROM, except thoracic flexion. Thoracic flexion: S.S. main effect of group x condition (F_1,21_ = 14.55). S.S. greater forward flexion post-SMT. S.S. main effect of group, condition and group x condition on every gait initiation variable. S.S. lower post-SMT than pre-SMT. N.S. change in sham group. *Unable to calculate mean change and 95% CI.
Learman et al., 2009 [41]	History of chronic low back pain with minimal to no pain at the time of testing; 18–65 years; USA; *n* = 33; Randomized, controlled, unbalanced crossover design, 1-week washout.	SMT: side-lying lumbar SMT at level of identified dysfunction. n = 33	Sham: side-lying position mimicking SMT held for 15 s. n = 33	Immediately and 1 week after intervention.	Trunk joint position sense (JPS), threshold to detect passive motion (TTDPM), direction of motion (DM) and force reproduction (FR).	JPS: S.S. period effect in SMT group (F = 3.026). 1-week residual effect: mean error reduction of 1.05° (98.33% CI = 0.16, 1.94). S.S. immediate treatment effect for sham group (t = 3.247). Mean error reduction: 0.82° (99% CI = 0.08°, 1.56°). TTDPM: S.S. group-period effect (F = 4.048, *p* = 0.013) SMT: 0.317° (98.33% CI = 0.04, 0.60) N.S. difference for DM or FR. *Unable to calculate mean change or 95% CI
Méndez-Sánchez et al. 2014 [14]	Asymptomatic men and women; 18–30 years; Spain; *n* = 62. Double-blind RCT.	Bilateral HVLA to SIJs plus placebo technique. *n* = 31	Placebo technique: mobilization without tension of the hips in the supine position. n = 31	Immediately after SMT.	Baropodometric analysis of surface (mm^2^), weight (kg) and percentage of load (%) on each forefoot, hindfoot and each foot in its entirety, and the location of the maximum pressure point on the plantar support.	Between group differences HVLA-placebo: Surface variable: Left foot: 0.06 mm^2^ (− 6.19, 6.31) Right foot: − 0.9 mm^2^ (− 8.96, 7.16) % of load: Left foot: 2.39% (− 0.15, 4.93) Right foot: − 2.39% (− 4.93, 0.15) Weight variable: Left foot: 1.84 kg (0.14, 3.54) Right foot: − 1.84 kg (− 3.54, − 0.14) Forefoot (FF) and Hindfoot (HF) measures: Surface variable: LFF: 0.93 mm^2^ (− 2.25, 4.11) RFF: 2.0 mm^2^ (− 2.45, 6.45) LHF: 1.71 mm^2^ (− 0.51, 3.93) RHF: 0.04 mm^2^ (− 2.83, 2.91) % of load: LFF: 0.32% (− 1.49, 2.13) RFF: − 0.39% (− 2.71, 1.93) LHF: 2.71% (0.53, 4.89) RHF: − 2.13% (− 3.79, − 0.47)
Puentedura et al., 2011 [43]	Healthy individuals from university faculty and students; 21–34 years; Las Vegas, US; *n* = 35; Single-blinded crossover RCT, 1-week washout.	Side-lying lumbar thrust joint SMT to the right side. n = 35	Sham: side-lying position Maitland grade I oscillation for lumbar rotation over 30 s bilaterally. n = 35	Immediate after condition.	Thickness of the transversus abdominus (TA) muscle during the abdominal drawing in maneuver (cm).	Between group differences SMT-Sham: Rest: − 0.014 cm (− 0.04, 0.01) Contracted: − 0.007 cm (− 0.05, 0.04)
Rosa et al., 2013 [44]	Asymptomatic; 19–28 years; Brazil; *n* = 55; Controlled before/after intervention laboratory study.	Seated thoracic SMT. *n* = 24	Sham: same position and procedure, without the high-velocity thrust. *n* = 21	Immediately after the intervention. SMT: n = 3 lost due to no cavitation	Disabilities of the Arm, Shoulder, and Hand Questionnaire (DASH), scapular plane abduction kinematics to determine scapulohumeral rhythm (GH:scapulothoracic ratio). Adverse events.	Between group differences, during different degrees of arm elevation: 30°-120°: 0.45 (− 0.79, 1.69) 30°-60°: − 0.05 (− 0.39, 0.29) 60°-90°: − 0.05 (− 0.15, 0.32) 90°-120°: 0.36 (− 0.11, 0.83) No adverse events.
Sport-Specific Outcomes						
Costa et al., 2009 [32]	Golfers with handicap 0 to 15, practicing at least 4 h 1x/week, 18–55 years; Brazil; *n* = 43; RCT.	Group 2: same standardized stretch program as Group 1 plus SMT. SMT provided to dysfunctional joints of the neck, thoracic spine and low back. 1x/week, 4 weeks. *n* = 23	Group 1: standardized stretch program. Static stretches were performed for 20 s bilaterally, including forearm flexors, deltoids, brachioradialis, biceps, forearm extensors, levator scapulae, gastrocnemius, soleus, quadriceps, hamstrings and gluteal muscles. *n* = 20	Immediately post intervention weekly.	Trial distance of 3 full swing maneuvers with driver club (average of 3 distances in meters).	Mean differences between groups (Group 2-Group 1): Immediately post-intervention week 1: 9.82 m (−3.58, 23.22) Immediately post-intervention week 2: 11.04 m (− 0.05, 22.13) Immediately post-intervention week 3: 4.39 m (− 5.54, 14.32) Immediately post-intervention week 4: 7.73 m (− 1.48, 16.94)
Humphries et al. 2013 [40]	Asymptomatic male recreational basketball players, completing at least 5/10 free throws; 16–37 years; Texas, USA; n = 24; RCT pilot study.	Left cervical SMT at C5/C6. *n* = 12	Sham: Activator set to zero force n = 12	Immediately after SMT.	Dominant handgrip isometric strength (kg) and free throw completion (20 free throws) (% completed). Adverse events.	Handgrip strength between SMT-placebo: 1.2 kg (−4.46, 6.86) Free throw accuracy between SMT-placebo: 2.4% (0.656, 4.14) No adverse events.
Olson et al., 2014 [42]	Asymptomatic cyclists; 29–43 years; Texas; n = 20; Blinded, randomized, crossover, controlled study, 1 week between interventions.	Condition A: bilateral HVLA side-posture SMT mammillary push at L3 with 15 min wait. n = 6	Condition B: 15 min bilateral sham acupuncture to arbitrary points on or near GB-34, SP-6, CV-6, Shenmen.*n* = 6 Control arm: no intervention. *n* = 8	15 min post intervention.	Sit and reach test (cm), time to completion of a 0.5 km cycle ergometer sprint against 4-kp resistance (sec), maximum exercise heart rate (bpm) and rate of perceived exertion (Borg 6–20 scale).	Between group differences HVLA-Sham Acupunctur: 0.5 km sprint time: 0.8 s (− 10.82, 12.42) Mean RPE: 0.2 (− 1.16, 1.56) Mean max HR: − 0.3 bpm (− 10.08, 9.48) Mean sit-and-reach test: − 0.6 cm (− 6.19, 4.99) N.S. training effect or test acclimation in the control group.
Sandell et al., 2008 [45]	Healthy, male junior running athletes training in middle distance; 17–20 years; Sweden; *n* = 17; Prospective, randomized, controlled experimental pilot study.	Side posture SIJ SMT, hip joint adjustment (prone posterior to anterior glide) chosen based on restrictions and same stretching program as control group. 1x/week for 3 weeks. n = 8	Control group: passive and active stretching, using hip flexor stretch, as part of their usual training activities. 2–3 times during the study period. *n* = 9	Within 3 days after 3-week intervention	Hip extension (°) and running velocity (30 m) (sec).	Hip extension between group differences SMT-Control: Right: − 3.8° (− 5.73, − 1.87) Left: − 2.9° (− 4.95, − 0.85) Running velocity between group differences SMT-Control: − 0.062 s (− 0.13, 0.002)

*AP* Anteroposterior, *ECG* Electrocardiogram, *FU* Follow-up, *HR* Heart rate, *HF* High frequency, *HVLA* High velocity low amplitude, *LF* Low frequency, *N.S*. Non-significant, *PA* Posteroanterior, *SIJ* Sacroiliac joint, *ROM* Range of motion, *SMT* Spinal manipulative therapy, *S.S*. Statistically significant, *VAS* Visual analog scale

### Statistical analyses

We computed the inter-rater reliability for each pair of phase 1 and phase 2 screening using the Cohen’s kappa coefficient (ĸ) [27, 28]. Where possible, the 95% confidence interval (CI) for the difference in mean change was calculated. We deemed a *p*-value of < 0.05 to be statistically significant.

### Reporting

This systematic review was organized and reported based on the Preferred Reporting Items for Systematic Review and Meta-Analyses (PRISMA) statement [29].

## Results

### Study selection

**Fig. 1:**
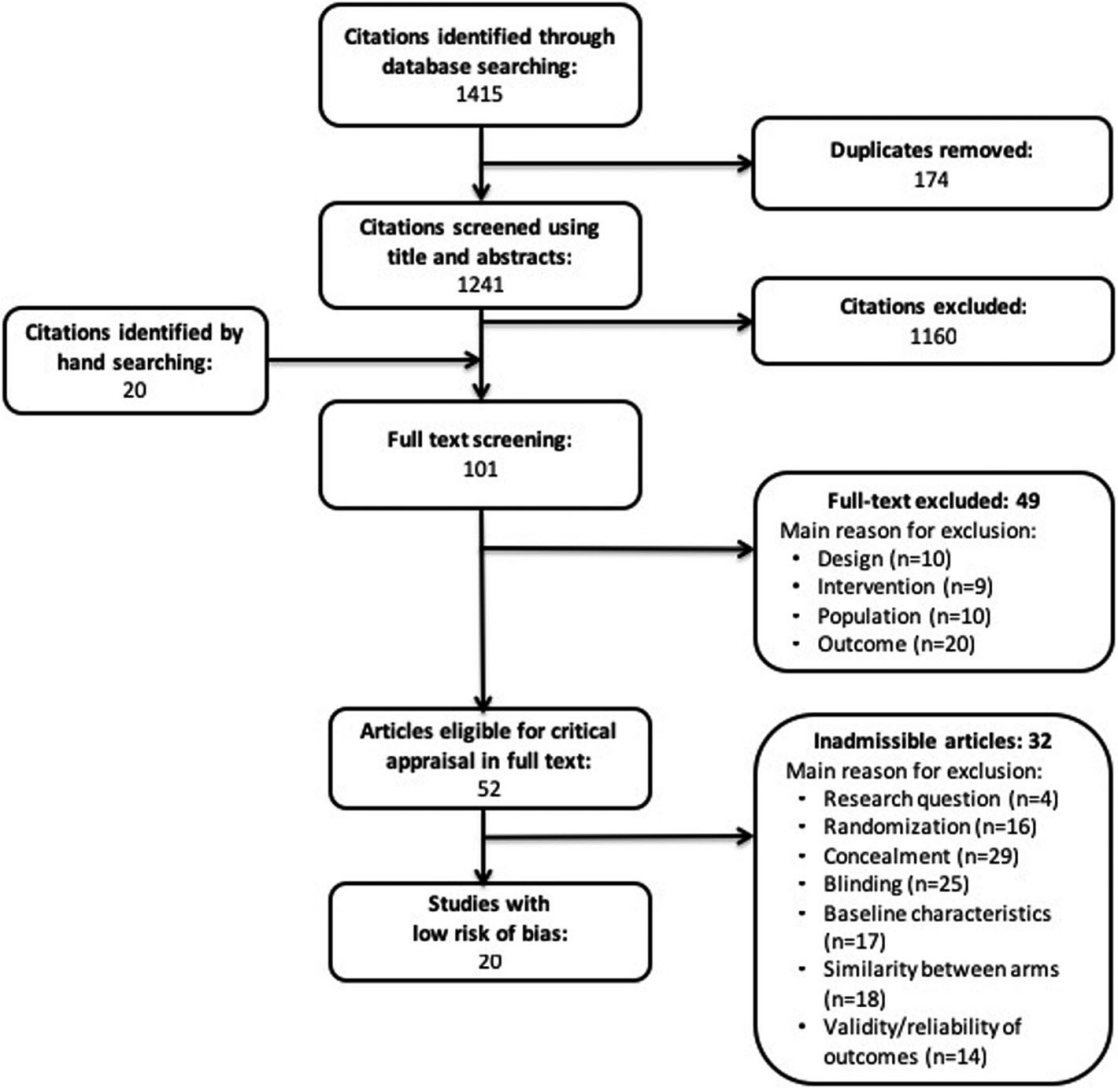
Flow diagram of study selection process

Our search retrieved 1415 articles, of which 52 articles were eligible for critical analysis after applying inclusion and exclusion criteria (Fig. 1). Articles were excluded after phase 2 screening due to ineligible study design, interventions not consistent with our definition of SMT, research questions addressing the effect of SMT on specific conditions and pain or the effect on SMT in asymptomatic adults with outcome measures not deemed to be relevant to performance (i.e. neurophysiological parameters, such as H-wave and M-wave).The mean interrater agreement for phase 1 was κ = 0.934 (range: 0.88–0.97) and phase 2 was κ = 0.5 (range 0.17–0.73). Disagreements were primarily related to outcome measures of screened studies due to the broad definition of performance-related outcomes. Disagreements were resolved with discussion between reviewing pairs. A total of 20 articles were deemed to have a low risk of bias and were included in our systematic review; articles deemed to have a high risk of bias were not included.

### Risk of bias within studies

All studies were appraised using the SIGN criteria for RCT, as none were cohort studies. Mean reliability of appraisal was κ = 0.39 (range: − 0.88-1.0). We critically appraised 52 studies, of these 32 were deemed to have a high risk of bias (Appendix 2) [see Additional file 2], and were not included in this review. Methodological weakness of the studies with a high risk of bias included lack of clear research question (*n* = 4), randomization (*n* = 16) or disclosure of concealment methodology (*n* = 29), lack of investigator or subject blinding (*n* = 25), no baseline characteristics of participants (*n* = 17), differences between groups at baseline (*n* = 17) and questionable validity and reliability of the outcome measures (*n* = 14) (Tables 2 and 3).

**Table 2:** Risk of bias table based on Scottish Intercollegiate Guidelines Network (SIGN) Criteria for high risk of bias studies; randomized controlled trials

Author, Year	Research Question	Randomization	Concealment	Blinding	Similarity at baseline	Similarity between arms	Outcome measures	Percent drop-out	Intention to treat	Results between sites	Level of evidence
Botelho et al., 2012	Y	Y	CS	N	N	N	Y	0%	NA	NA	1-
Cardinale et al., 2015	Y	Y	N	CS	Y	Y	CS	0%	NA	NA	1-
Enebo et al., 2003	Y	Y	CS	N	CS	CS	CS	0%	CS	NA	1-
Engel et al. 2007	Y	Y	Y	CS	N	N	CS	20%	N	NA	1-
Fox et al., 2006	Y	Y	CS	N	Y	Y	CS	CS	CS	NA	1-
Miller et al., 2000	Y	Y	N	N	Y	Y	CS	CS	Y	NA	1-
Nansel et al., 1992	Y	Y	CS	N	CS	CS	Y	0%	Y	NA	1-
Nansel et al., 1993	Y	Y	CS	Y	CS	Y	CS	0%	Y	NA	1-
Palmgren et al., 2009	Y	Y	Y	N	CS	Y	Y	0%	Y	NA	1-
Passmore et al., 2010	Y	Y	CS	Y	CS	CS	Y	0%	Y	NA	1-
Pollard et al., 1998	Y	Y	CS	CS	CS	CS	CS	CS	CS	NA	1-
Schwartzbauer et al., 1997	CS	Y	N	N	CS	CS	CS	25%	Y	NA	1-
Shrier et al., 2006	Y	Y	CS	CS	Y	Y	Y	17.6%	Y	NA	1-
Stamos-Papastamos et al., 2011	Y	Y	CS	N	Y	Y	Y	CS	Y	NA	1-
Straub et al., 2001	Y	Y	CS	CS	CS	CS	Y	0%	Y	NA	1-
Ward et al., 2012	N	Y	N	N	CS	CS	CS	5%	NA	NA	1-
Ward et al., 2013	Y	Y	Y	N	N	N	Y	0%	CS	NA	1-
Ward et al., 2014	Y	Y	N	N	Y	Y	N	0%	Y	NA	1-

*Y* Yes, *N* No, *CS* Can’t say, *NA* Not applicable

**Table 3:** Risk of bias table based on Scottish Intercollegiate Guidelines Network (SIGN) Criteria for high risk of bias studies; non-randomized trials

Author, Year	Research Question	Randomization	Concealment	Blinding	Similarity at baseline	Similarity between arms	Outcome measures	Percent drop-out	Intention to treat	Results between sites	Level of evidence
Barbosa et al., 2014	Y	N	NA	NA	CS	CS	Y	0%	Y	NA	1-
Bonci et al., 1990	N	N	NA	NA	Y	N	CS	0%	Y	NA	1-
Deutschmann et al., 2011	Y	N	NA	NA	Y	N	Y	0%	Y	NA	1-
Lauro et al., 1991	Y	CS	NA	NA	N	CS	CS	CS	Y	NA	1-
Nansel et al., 1991	Y	N	NA	NA	CS	CS	Y	0%	Y	NA	1-

*Y* Yes, *N* No, *CS* Can’t say, *NA* Not applicable

According to SIGN Criteria, if groups are not randomized, the criteria can continue to be used, but sections 1.2, 1.3, and 1.4 are not applied, corresponding to randomization, concealment and blinding

The 20 articles accepted [13, 14, 30–47] for this review were deemed to have a low risk of bias. Methodological weaknesses in these studies included no disclosure of concealment methodology (*n* = 12), lack of participant or investigator blinding (*n* = 7), similarities at baseline (n = 1) (Table 4). We calculated between group mean change and 95% CI for all but four studies [33, 34, 38, 41].

**Table 4:** Risk of bias table based on Scottish Intercollegiate Guidelines Network (SIGN) Criteria for low risk of bias studies

Author, Year	Research Question	Randomization	Concealment	Blinding	Similarity at baseline	Similarity between arms	Outcome measures	Percent drop-out	Intention to treat	Results between sites	Level of evidence
Budgell B and Polus B, 2006	Y	Y	N	CS	Y	Y	Y	10.7%	Y	NA	1+
Christiansen et al., 2018	Y	Y	CS	N	Y	Y	Y	8%	Y	NA	1+
Costa et al., 2009	Y	Y	CS	Y	Y	Y	Y	0%	Y	CS	1+
Da Silva et al., 2013	Y	Y	CS	Y	N	Y	Y	6%	Y	NA	1+
Ditcharles et al., 2017	Y	Y	Y	N	Y	Y	Y	0%	Y	NA	1+
Dunning et al., 2009	Y	Y	CS	N	Y	Y	Y	0%	NA	NA	1+
Galindez-Ibarbengoetxea et al., 2017	Y	Y	Y	Y	Y	Y	Y	0%	Y	NA	1+
Gavin D, 1999	Y	Y	CS	Y	Y	Y	Y	0%	Y	NA	1+
Grindstaff et al., 2009	Y	Y	CS	CS	Y	Y	Y	0%	Y	NA	1+
Hanney et al., 2017	Y	Y	Y	N	Y	Y	Y	0%	Y	NA	1+
Humphries et al. 2013	Y	Y	CS	Y	Y	Y	Y	0%	Y	NA	1++
Learman et al., 2009	Y	Y	CS	N	Y	Y	CS	0%	Y	NA	1+
Mendez-Sanchez et al. 2014	Y	Y	CS	Y	Y	Y	Y	0%	Y	NA	1++
Olson et al., 2014	Y	Y	Y	Y	Y	Y	Y	0%	Y	NA	1++
Pollard et al., 1996	Y	Y	CS	Y	Y	Y	Y	0%	Y	NA	1+
Puentedura et al., 2011	Y	Y	Y	Y	CS	Y	Y	0%	Y	NA	1+
Rosa et al., 2013	Y	Y	Y	Y	Y	Y	Y	14.2%	Y	NA	1+
Sandell et al., 2008	Y	Y	Y	Y	CS	Y	Y	0%	Y	NA	1+
Sanders et al., 2015	Y	Y	Y	Y	Y	Y	Y	0%	Y	NA	1++
Ward J, 2013	Y	Y	N	Y	Y	Y	Y	0%	Y	NA	1+

*Y* Yes, *N* No, *CS* Can’t say, *NA* Not applicable

### Study characteristics

We included 20 articles with a low risk of bias, of which seven were randomized controlled crossover studies [30, 31, 35, 41–43, 46], 10 were randomized controlled trials (RCT) [13, 14, 32–34, 37–39, 44, 47] and three were RCT pilot studies [36, 40, 45].

Location, type and direction of SMT, and outcome measures (physiological, biomechanical or sport-specific) varied in each study (Table 1). Studies included had various theoretical frameworks for the location and direction of SMT; including neurophysiological, spinal restriction or dysfunction, and theoretical associations of segment dysfunction with specific outcomes. Four studies reported on adverse events [30, 36, 40, 44]. Four studies reported on physiological outcomes [30, 33, 42, 47], 16 studies reported on biomechanical outcomes [13, 14, 31, 34–46], three of which also reported on sport-specific outcomes [40, 42, 45], and one additional study reported on performance variables for specific sports [32, 42].

### Physiological outcomes

Four studies investigated the effect of SMT on physiological outcomes in asymptomatic subjects [30, 33, 42, 47] (Table 1). There was no significant effect of thoracic SMT on resting heart rate (HR) or HR variability and no effect of lumbar SMT on exercising HR [30, 42, 47]. In addition, Ward [47] showed that mid-lumbar SMT had no significant effect on other exercise science measures, including rate of perceived exertion (RPE) during the Bruce treadmill test, calculated VO_2_max, or blood lactate concentration [47]. This was further supported by Olson et al. [42] where they reported no significant effect of bilateral mid-lumbar SMT on RPE during a 500 m cycle ergometer sprint [42]. In the fourth study, Da Silva et al. [33] examined the effect of cervical, thoracic, combination SMT (cervical and thoracic) and extremity mobilization (placebo group) on maximum inspiratory pressure (MIP), maximum expiratory pressure (MEP) and total lung capacity (TLC) in healthy University students [33]. There were no significant between-group differences reported and we did not calculate mean change and 95% CI from this study [33].

### Biomechanical outcomes

#### Electromyographical/muscle force

Seven studies examined the effect of SMT on muscle activation or strength of the lower limb in healthy subjects [13, 31, 35, 36, 38, 40, 46] (Table 1). Four of these studies identified a significant change in muscle function [31, 35, 36, 38]. SMT based on identified joint dysfunction of the cervical, thoracic and lumbar spine, and pelvis was shown to increase soleus plantar flexion maximum voluntary contraction (MVC) compared to a control group of similar active and passive range of motion (ROM) [31]. These increases were statistically significant immediately and 30 min after SMT but not 60 min after [31]. In a study comparing lumbopelvic SMT to passive ROM and passive prone extension interventions, quadriceps MVC and activation immediately significantly increased from baseline [38]. These differences were transient and were not statistically significant between the three groups at later time points (i.e. 20, 40 or 60 min) [38]. We did not calculate mean change and 95% CI from this study. In contrast, two studies reported no significant effect of SMT; one showed no significant change in quadriceps MVC after L3–4 SMT compared to sham manipulation [13] and the second showed no significant change in knee extension or flexion muscle activation or peak torques at 60°/sec and 180°/sec during isokinetic concentric testing compared to a non-specific drop piece procedure [46].

Three studies investigated the effect of cervical SMT on muscle force or function immediately post intervention [35, 36, 40] (Table 1). The first study compared cervical SMT based on identified joint dysfunction, to SMT to the right C5 segment, and passive range of motion (ROM) groups [36]. There was no significant difference between groups for peak cervical flexion isometric contraction. There was no significant difference in resting biceps EMG except for the left biceps with SMT based on joint dysfunction or C5 segment compared to the ROM group. There was no significant difference in sternocleidomastoid (SCM) muscle activation during the cranio-cervical flexion test except for the right SCM in the manipulation by dysfunction group compared to the ROM group [36]. Humphries et al. [40] found no significant effect of left cervical C5–6 SMT on right handgrip strength compared to an Activator adjusting instrument set to zero force [40]. In contrast, Dunning et al. [35] identified that right-sided cervical SMT to C5–6 segment significantly increased bilateral biceps brachii resting EMG activity greater than sham SMT to C5–6 segment or no manual contact [35]. This effect was significantly greater on the right, compared to the left side [35].

#### Range of motion

Two studies identified specific ROM changes immediately after thoracic SMT in asymptomatic healthy subjects [34, 37] (Table 1). Seated or supine thoracic SMT to dysfunctional segments between T3–8 significantly increased bilateral lateral flexion when SMT was compared to the control group [37]. There was no significant difference in forward flexion or in lateral flexion when comparing SMT to the palpation group [37]. In the second study, SMT to T9 resulted in significant increased thoracic forward flexion compared to a sham procedure (SMT setup with no thrust), but no significant changes in thoracic extension, lumbar flexion or lumbar extension [34]. However, we did not calculate mean change and 95% CI of this study.

Two studies examined the effect of cervical spine SMT on ROM [36, 39]. Bilateral cervicothoracic SMT significantly increased cervical ROM compared to an upper trapezius stretch and no intervention; however, differences varied based on the specific ROM (Table 1) (see Additional file 1) [39]. For all ranges except extension and right cervical rotation, there were significant differences between SMT and control groups [39]. For right lateral flexion, cervical flexion and left cervical rotation there were also significant differences between control and stretch groups [39]. For cervical extension, there was an increase in ROM when comparing SMT to stretch group and stretch to control group, but not between SMT and the control group [39]. For right cervical rotation, there were no significant changes [39]. In a study comparing cervical SMT based on joint dysfunction, to right sided C5 SMT and passive ROM groups, there were no significant changes in range of motion except for a significant increase in extension in both SMT groups compared to the passive ROM group and an increase in right lateral flexion with manipulation based on dysfunction compared to the passive ROM group [36].

Two studies investigated lumbopelvic SMT and ROM [42, 45] (Table 1). The first found that there was no significant effect of bilateral mid-lumbar SMT on the sit-and-reach test compared to a sham manipulation [42]. The second found that three weeks of weekly sacroiliac joint (SIJ) SMT, hip manipulation and a stretching program did not significantly increase hip extensibility, as measured with the modified Thomas Test, compared to a stretching program alone [45].

#### Baropodometric and gait parameters

Two studies examined the effect of SMT on baropodometry [14, 34] (Table 1). Mendez-Sanchez et al. [14] showed that bilateral SIJ SMT resulted in a significant change the weight variable (kg) of both feet and the % load of both hind feet compared to placebo technique of hip mobilization with no tension [14]. Ditcharles et al. [34] examined the effect of T9 SMT on gait initiating parameters [34]. They reported a significant decrease in all parameters measured, including anticipatory postural adjustments (APA) duration, peak of anticipatory backward center of pressure (COP) displacement, center of gravity (COG) velocity at toe-off (TO), mechanical efficiency of APA, peak of COG velocity, step length, and swing phase duration, immediately after SMT compared to controls (SMT setup with no thrust) [34]. Unfortunately, we did not calculate mean change and 95% CI from this study.

#### Other biomechanical outcomes

Additional biomechanical outcomes reported in studies included lumbar spine proprioception, transversus abdominis (TA) thickness and scapular kinematics. Learman et al. [41] showed significant improvements in lumbar spine trunk joint position sense (JPS) in a randomized AB:BA crossover trial [41]. For the group that received SMT on the first session, JPS had an immediate significant improvement and a one-week residual effect [41]. In contrast, while the group that had a side-lying sham on the first session had a significant immediate improvement, there was no residual effect [41]. JPS was defined as the ability of a participant to reproduce trunk angle from neutral, after being passively brought to a position [41]. However, we did not measure mean change and 95% CI from this study. Lumbar SMT had no significant effect on TA thickness measured with multiple ultrasound readings at rest or during contraction compared to a sham procedure [43]. TA thickness is used as an indicator of muscle activation [43]. Thoracic SMT did not have a significant immediate effect on scapular kinematics or scapulohumeral rhythm measured with 3D kinematics [44].

### Performance-based outcomes

Two studies examined the immediate effect of a single session of SMT on sport-specific parameters but found no differences between groups [40, 42] (Table 1). Humphries et al. [40] found that left-sided C5–6 SMT had an immediate small significant effect on basketball free-throw accuracy compared to a sham procedure of 2.4% ((95%CI (0.656, 4.14)) [40]. The sham procedure was an instrument-assisted intervention (Activator) set to provide zero force [40]. Olson et al. [42] found no significant effect of bilateral lumbar SMT on 0.5 km cycling sprint time compared to acupuncture and control groups within 15 min post intervention [42].

In addition, two studies assessed a course of weekly SMT on sport-specific parameters [32, 45]. Costa et al. [32] compared four weeks of weekly full spine SMT based on identified restriction and a standardized stretch program to the standardized stretch program alone on a golfing full-swing range [32]. There were no significant differences between groups [32]. Similarly, Sandell et al. [45] found that three weeks of weekly SIJ SMT, hip manipulation and stretching program had no significant effect on maximum running velocity over a 30 m distance after a 30 m running start, compared to a stretching program alone [45].

### Adverse events

Four studies reported on adverse events [30, 36, 40, 44]. Of these, three studies reported no adverse events with any aspect of their protocol, including SMT [36, 40, 44]. Budgell and Polus [30] reported one subject in the sham group developed pain (increase in visual analogue scale (VAS) to 3.8/10) after the intervention, and two subjects in the SMT group developed pain (increase in VAS to 1.3 and 1.4/10) after the intervention [30].

## Discussion

### Key findings

The preponderance of evidence suggests that SMT in comparison to sham or other interventions does not significantly enhance performance-based outcomes as we defined. Several studies did report significant immediate effects of SMT in quadriceps and ankle plantarflexion MVC [31, 38], resting biceps brachii EMG [35], and trunk joint position sense [41]. It is unclear what impact these effects may have upon enhancing human performance over longer periods. However, there is one low risk of bias study suggesting that a single SMT improves basketball free throw accuracy immediately post intervention compared to sham (2.4%, 0.66–4.14), which may be relevant in high-level sport performance [32]. Only two studies provided more than one SMT over a time [37, 42], neither which had a significant effect but broad CI suggest potential issue with sample size.

Other statistically significant changes were noted in ROM [34, 36, 37, 39] and baropodometric variables [14, 34]; however, the importance of such changes in uncertain. First, both these studies measured numerous ROM and additional outcomes and could be statistically significant based on chance alone. Second, in studies with a potentially minimally important change, the 95% CI were wide, indicating imprecision of the outcome [36]. Conversely, in studies with narrow 95% CI [37, 39], the noted significant changes may be of questionable relevant importance, especially in the cervical spine where results were less than reported measurement range of smallest detectable difference of 10–19° [48].

Only four of 20 included studies reported adverse events. Of these studies, only minor adverse events were reported, which were associated with both SMT and sham procedures. These were described as increased pain after the intervention. The increases in VAS, particularly in the SMT group, may not be clinically significant, as the median VAS to denote a clinically important change in acute pain is 1.7 [49]. No further details on management of these adverse events were provided.

### Updating current literature

The present systematic review provides an update to previous reviews by including articles investigating the effect of SMT in asymptomatic adult populations rather than athletes alone. The systematic review by Cerqueira et al. [11] examined the effect of HVLA SMT and athletic performance and found only five relevant studies [11]. Only two of these studies were included in our systematic review [40, 45], as two others were critically appraised but deemed to have a high risk of bias [50, 51], and one was not retrieved by our search strategy as the intervention was a HVLA thrust to the tibiotarsal joint, not the spine. Botelho et al. [12] conducted a similar systematic review and included seven relevant studies [12], four of which overlapped with Cerquira et al. [11] and four included in our systematic review. The three studies not included in our review were deemed to have a high risk of bias [50–52]. Both these recent reviews concluded that the current evidence is insufficient to determine if SMT should be used to improve athletic performance and future high quality research is required [11, 12].

Our systematic review suggests that SMT compared to sham or other interventions does not enhance performance-based outcomes as we defined, except in a few areas. The relevance to of such changes to improvement in functional or sport performance is unclear at this time. We agree with Cerqueira et al. [11] and Botelho et al. [12], that studies in this field require greater methodological rigor. Specifically, studies exploring athletic performance and the effect of SMT in asymptomatic adults should focus on identifying relevant and responsive outcome measures, use adequate sample sizes, include appropriate control groups, assess outcomes over longer temporal intervals, and explore the role of athlete expectations prior to intervention.

These recommendations are highlighted by various trends in this systematic review. Outcome measures varied widely across studies, often lacking reporting of measurement properties and appropriateness of these tools for the desired outcome. The longest time interval for the effect of SMT was one week by Learman et al. [41], which included a one week washout period in the crossover study [41]. Otherwise, the longest follow-up was 60 min after SMT [31, 38]. The rationale for SMT in each study varied, including SMT to identified joint dysfunction [31, 36], neurophysiological association of spinal segment and associated muscle groups [31, 38, 46], and SMT to T9 due to its classic definition as the “walking vertebra” [34]. This questions the validity and reliability of some theories, such as those historically based with limited scientific rationale or the reliability of identifying of spinal restrictions [53, 54]. Finally, 52 studies were eligible for full text appraisal; however, 32 were deemed to have a high risk of bias, indicating the overall poor methodological quality of this body of literature.

The vast majority of studies included in our review are exploratory in nature; they assess interventional efficacy, collect short-term outcomes, and can be used to design evaluation studies providing evidence for effectiveness [25]. We identified two studies that could be considered evaluation studies [32, 45]; however, when mean change and 95% CI were measured, there was no effect of SMT on full-swing golfing range or running velocity, and a small mean change (2.9°-3.9°) on hip extensibility [32, 45]. In addition, the included studies use different theories and outcomes that provides limited guidance in designing appropriate evaluation studies.

### Strengths and limitations

Our systematic review has several strengths. These include a search strategy that was developed and checked through peer review and adapted for a broad set of databases to ensure identification of all possibly relevant articles. We also expanded our search terms to not only include athletes or sport performance, but to include studies that would have implications to athletes by including all asymptomatic adults. We used two independent reviewers for screening and critical appraisal to minimize error and bias and used a well-accepted and valid set of criteria (SIGN) for the critical appraisal of relevant studies.

This systematic review also has limitations. We did not perform a sensitivity analysis on thresholds for low risk of bias studies. The use of certain definitions in our research question was broad, such as performance-related outcomes, which may have made it difficult to identify relevant articles during the screening process, as evidenced by a large range of kappa-values. In addition, we may have missed studies that have secondary outcomes relevant to performance-outcomes that were not included in the title or abstract. We tried to mitigate such losses by hand searching relevant studies, but we did not search systematic reviews. We restricted our search to studies published in the English language, which may have excluded relevant studies. However, previous reviews have found that this has not led to biases in the reported results. [55]

## Conclusion

In conclusion, the preponderance of evidence suggests that SMT compared to sham or other intervention does not enhance performance-related outcomes. Exceptions include exploratory evidence suggesting improvements in ankle plantarflexion and quadriceps MVC, resting biceps brachii EMG, lumbar joint position sense and basketball free throw accuracy. These findings are consistent with neurophysiological studies, wherein evidence suggests that SMT affects reflex responses at the spinal and cortical levels [56, 57]. We found no conclusive explanatory evidence that SMT affects performance-related outcomes in the asymptomatic adult population. Further high-quality performance specific studies are required to confirm these preliminary findings.

## Additional files

Additional file 1: **Appendix I.** Search strategy and search terms. (DOCX 15 kb)

Additional file 2: **Appendix II.** References for high risk of bias studies not included in this review. (DOCX 17 kb)

## Abbreviations

APA: Anticipatory postural adjustments
COG: Center of gravity
COP: Center of pressure
EMG: Electromyography
HR: Heart rate
MEP: Maximal expiratory pressure
MIP: Maximal inspiratory pressure
MVC: Maximal voluntary contraction
RCT: Randomized controlled trial
ROM: Range of motion
SIJ: Sacroiliac joint
SMT: Spinal manipulative therapy
TA: Transversus abdominis
TLC: Total lung capacity
TO: Toe-off
