# Supplementary material for: The effects of spinal manipulation on performance-related outcomes in healthy asymptomatic adult population: a systematic review of best evidence
Source: Chiropr Man Therap. 2019 Jun 7;27:25. doi: 10.1186/s12998-019-0246-y (PMC6555009; doi:10.1186/s12998-019-0246-y)
Supplement: Supplementary file 1 — Appendix I. Search strategy and search terms. (DOCX 15 kb) [file 12998_2019_246_MOESM1_ESM.docx]

**Appendix I: Search Strategy**

Population: asymptomatic adult individuals, including athletic and non-athletic populations.

Intervention: spinal manipulative therapy (including the cervical, thoracic, lumbar spine, and sacroiliac joints).

Comparators: other interventions, placebo/sham interventions, no intervention.

Outcomes: performance-related outcomes or clinically measurable changes that may impact performance. This includes physiological, biomechanical and sport-specific outcomes. Pain was not included as this would not be present in asymptomatic populations. Pain pressure threshold was not included as connection to performance is not clear.

1. (MH "Chiropractic")
2. (MH "Manipulation, Spinal")
3. (MH "Musculoskeletal Manipulations")
4. (MH "Manipulation, Chiropractic")
5. chiroprac*
6. TI spinal manip* or AB spinal manip*
7. TI manual therap* or AB manual therap*
8. hvla
9. high velocity low amplitude
10. or 1-9
11. (MH "Athletic Performance")
12. (MH "Joints")
13. (MH "Postural Balance")
14. (MH "Hand Strength")
15. (MH "Muscle Strength")
16. (MH "Sports+")
17. TI performance* or AB performance*
18. (MH "Musculoskeletal Physiological Phenomena+")
19. (MH "Muscle Strength Dynamometer")
20. (MH "Biomechanical Phenomena")
21. TI wrestling or AB wrestling or TI wrestler* or AB wrestler*
22. TI weight lift* or AB weight lift*
23. TI walking or AB walking
24. TI volleyball or AB volleyball
25. TI (track n2 field) or AB (track n2 field)
26. TI diving or AB diving or TI diver or AB diver or TI divers or AB divers
27. TI swim* or AB swim*
28. TI soccer or AB soccer
29. TI skiing or AB skiing or TI skier* or AB skier*
30. TI skating or AB skating or TI skater* or AB skater*
31. TI jogging or AB jogging or TI jogger or AB jogger
32. TI runner* or AB runner*
33. TI running or AB running
34. TI tennis or AB tennis
35. TI racquet* or AB racquet*
36. TI mountaineer* or AB mountaineer*
37. TI tai ji or AB tai ji
38. TI martial arts or AB martial arts
39. TI hockey or AB hockey
40. TI sport* or AB sport*
41. TI gymnast* or AB gymnast*
42. TI golf* or AB golf*
43. TI football or AB football
44. TI boxing or AB boxing or TI boxer* or AB boxer*
45. TI cycling or AB cycling or TI cyclist* or AB cyclist*
46. TI basketball or AB basketball
47. TI baseball or AB baseball
48. TI athlet* or AB athlet*
49. TI judo or AB judo
50. TI rugby or AB rugby
51. TI neuromusc* or AB neuromusc*
52. TI joint* or AB joint*
53. TI biomechanic* or AB biomechanic*
54. TI balance or AB balance
55. reaction time*
56. agility
57. TI speed or AB speed
58. TI power or AB power
59. TI movement* or AB movement*
60. motor control*
61. motor pattern*
62. TI strength* or AB strength*
63. TI mobilit* or AB mobilit*
64. TI range or AB range
65. "range of motion"
66. or 11-65
67. asymptomat*
68. non-injured or non injured or noninjured
69. injury free or injury-free or injuryfree
70. pain free or pain-free or painfree
71. TI healthy or AB healthy
72. Or 67-71
73. 10 and 66 and 72
74. Limit 73 to English and French and 1990 to the present
