# Supplementary material for: The effects of spinal manipulation on performance-related outcomes in healthy asymptomatic adult population: a systematic review of best evidence
Source: Chiropr Man Therap. 2019 Jun 7;27:25. doi: 10.1186/s12998-019-0246-y (PMC6555009; doi:10.1186/s12998-019-0246-y)
Supplement: Supplementary file 2 — Appendix II. References for high risk of bias studies not included in this review. (DOCX 17 kb) [file 12998_2019_246_MOESM2_ESM.docx]

**Appendix II: References for high risk of bias studies**

1. Barbosa AWC, Silva AM, Silva AF, Martins FLM, Almeida Barbosa MCS. Immediate improvements in activation amplitude levels of the deep abdominal muscle following a sacroiliac joint manipulation during rapid upper limb movement. J Bodyw Mov Ther. 2014;18(4):626–32.
2. Bonci A, Ratliff R. Strength modulation of the biceps brachii muscles immediately following a single manipulation of the C4/5 intervertebral motor unit in healthy subjects; Preliminary report. Am J Chin Med. 1990;5(1):14–8.
3. Botelho MB, Andrade BB. Effect of cervical spine manipulative therapy on judo athletes’ grip strength. J Manipulative Physiol Ther. 2012;35(1):38–44.
4. Campbell BD, Snodgrass SJ. The effects of thoracic manipulation on posteroanterior spinal stiffness. J Orthop Sports Phys Ther. 2010 Nov;40(11):685–93.
5. Cardinale M, Boccia G, Greenway T, Evans O, Rainoldi A. The acute effects of spinal manipulation on neuromuscular function in asymptomatic individuals: A preliminary study. Phys Ther Sport. 2015 May;16(2):121–6.
6. Deutschmann K, Jones A, Korporaal C. A non-randomised experimental feasibility study into the immediate effect of three different spinal manipulative protocols on kicking speed performance in soccer players. Chiropr Man Therap. 2015;23(1):1.
7. Enebo BA. The effect of cervical spine manipulation on motor control in healthy individuals: A pilot study. Chiropr J Aust. 2013;33(3):93–7.
8. Engel RM, Vemulpad S. The Effect of Combining Manual Therapy with Exercise on the Respiratory Function of Normal Individuals: A Randomized Control Trial. J Manipulative Physiol Ther. 2007;30(7):509–13.
9. Fox M. Effect on hamstring flexibility of hamstring stretching compared to hamstring stretching and sacroiliac joint manipulation. Clin Chiropr. 2006;9(1):21–32.
10. Grassi D de O, de Souza MZ, Ferrareto SB, Montebelo MI de L, Guirro EC de O. Immediate and lasting improvements in weight distribution seen in baropodometry following a high-velocity, low-amplitude thrust manipulation of the sacroiliac joint. Man Ther. 2011 Oct;16(5):495–500.
11. Haavik H, Murphy B. Subclinical neck pain and the effects of cervical manipulation on elbow joint position sense. J Manipulative Physiol Ther. 2011 Feb;34(2):88–97.
12. Lauro A, Mouch B. Chiropractic effects on athletic ability. J Chiropr Res Clin Investig. 1991;6(4):84-.
13. Marshall P, Murphy B. The Effect of Sacroiliac Joint Manipulation on Feed-Forward Activation Times of the Deep Abdominal Musculature. J Manipulative Physiol Ther. 2006;29(3):196–202.
14. Mello MS, Pagnez MA, Cabral R de S, Taciro C, Nogueira LAC. Occipitoatlantoaxial Manipulation for Immediate Increase to Cervical Rotation. Altern Ther Health Med. 2016;22(2):18–22.
15. Miller JA, Bulbulian R, Sherwood WH, Kovach M. The effect of spinal manipulation and soft tissue massage on human endurance and cardiac and pulmonary physiology - A pilot study. Sport Chiropratic Rehabil. 2000;14(1):11–5.
16. Nansel D, Jansen R, Cremata E, Holley D. Effects of cervical adjustments on lateral-flexion passive end-range asymmetry and on blood pressure, heart rate and plasma catecholamine levels. J Manipulative Physiol Ther. 1991;14(8):450–6.
17. Nansel D, Peneff A, Quitoriano J. Effectiveness of upper versus lower cervical adjustments with respect to the amelioration of passive rotational versus lateral-flexion end-range asymmetries in otherwise asymptomatic subjects. J Manipulative Physiol Ther. 1992;15(2):99–105.
18. Nansel D, Peneff A, Cremata E, Carlson J. Time course considerations for the effects of unilateral lower cervical adjustments with respect to the amelioration of cervical lateral-flexion passive end-range asymmetry. J Manipulative Physiol Ther. 1993;13(6):297–304.
19. Nansel D, Waldorf T, Cooperstein R. Effect of cervical spinal adjustments on lumbar paraspinal muscle tone: Evidence for facilitation of intersegmental tonic neck reflexes. J Manipulative Physiol Ther. 1993;16(2):91–5.
20. Nougarou F, Page I, Loranger M, Dugas C, Descarreaux M. Neuromechanical response to spinal manipulation therapy: effects of a constant rate of force application. BMC Complement Altern Med. 2016;16(161):2–9.
21. Palmgren PJ, Lindeberg A, Nath S, Heikkilä H. Head Repositioning Accuracy and Posturography Related to Cervical Facet Nerve Blockade and Spinal Manipulative Therapy in Healthy Volunteers: A Time Series Study. J Manipulative Physiol Ther. 2009;32(3):193–202.
22. Passmore SR, Burke JR, Good C, Lyons JL, Dunn AS. Spinal manipulation impacts cervical spine movement and fitts’ task performance: a single-blind randomized before-after trial. J Manipulative Physiol Ther. 33(3):189–92.
23. Pollard H, Ward G. The effect of sacroiliac manipulation on hip flexion range of motion. Australas Chiropr Osteopathy. 1997;6(3):80–4.
24. Schwartzbauer J, Kolber J, Schwartzbauer M, Hart J, Zhang J. Athletic performance and physiological measures in baseball players following upper cervical chiropractic care: A pilot study. J Vertebr subluxation Res. 1997;1(4):33–9.
25. Shrier I, Macdonald D, Uchacz G. A pilot study on the effects of pre-event manipulation on jump height and running velocity. Br J Sports Med. 2006;40:947–9.
26. Stamos-Papastamos N, Petty NJ, Williams JM. Changes in bending stiffness and lumbar spine range of movement following lumbar mobilization and manipulation. J Manipulative Physiol Ther. 2011 Jan;34(1):46–53.
27. Straub WF, Spino MP, Alattar MM, Pfleger B, Downes JW, Belizaire MA, et al. The effect of chiropractic care on jet lag of Finnish junior elite athletes. J Manipulative Physiol Ther. 2001;24(3):191–8.
28. Surkitt D, Gibbons P. High velocity low amplitude manipulation of the atlanto-axial joint: Effect of atlanto-axial and cervical spine rotation asymmetry in asymptomatic subjects. J Osteopath Med. 2000;3(1):13–9.
29. Ward JS, Coats J, Ramcharan M, Humphries K, Tong T, Chu C. Thoracolumbar spinal manipulation and the immediate impact on exercise performance. J Chiropr Med. 2012;11(4):233–41.
30. Ward JS, Coats J, Sorrels K, Walters M, Williams T. Pilot study of the impact sacroiliac joint manipulation has on walking kinematics using motion analysis technology. J Chiropr Med. 2013;12(3):143–52.
31. Ward J, Sorrels K, Coats J, Pourmoghaddam A, Deleon C, Daigneault P. Pilot study of the impact that bilateral sacroiliac joint manipulation using a drop table technique has on gait parameters in asymptomatic individuals with a leg length inequality. J Can Chiropr Assoc. 2014 Mar;58(1):85–95.
32. Welch A, Boone R. Sympathetic and parasympathetic responses to specific diversified adjustments to chiropractic vertebral subluxations of the cervical and thoracic spine. J Chiropr Med. 2008;7(3):86–93.
